# Human–AI Interaction in Interventional Radiology: A Narrative Review of Current Applications, Challenges, and Future Directions

**DOI:** 10.3390/jimaging12060274

**Published:** 2026-06-22

**Authors:** Francesco Mariotti, Laura Maria Cacioppa, Nicolo’ Rossini, Alessandra Bruno, Giangabriele Francavilla, Alessandro Felicioli, Marco Macchini, Andrea Coppola, Michaela Cellina, Chiara Floridi

**Affiliations:** 1 Department of Clinical, Special and Dental Sciences, University Politecnica delle Marche, 60126 Ancona, Italy; dottfrancescomariotti@gmail.com (F.M.); a.bruno@pm.univpm.it (A.B.); gianghifrancavilla@gmail.com (G.F.); c.floridi@staff.univpm.it (C.F.); 2 Division of Interventional Radiology, Department of Radiological Sciences, University Hospital “Azienda Ospedaliero Universitaria delle Marche”, 60126 Ancona, Italy; nicolorossini44@gmail.com (N.R.); alessandro.felicioli@ospedaliriuniti.marche.it (A.F.); marco.macchini@ospedaliriuniti.marche.it (M.M.); 3 Division of Radiology, Department of Radiological Sciences, University Hospital “Azienda Ospedaliero Universitaria delle Marche”, 60126 Ancona, Italy; 4 Diagnostic and Interventional Radiology Unit, Circolo Hospital, ASST Sette Laghi, 21100 Varese, Italy; andrea.coppola@asst-settelaghi.it; 5 Department of Medicine and Technological Innovation, Insubria University, 21100 Varese, Italy; 6 Radiology Department, ASST Fatebenefratelli Sacco, 20121 Milan, Italy; michaela.cellina@asst-fbf-sacco.it

**Keywords:** interventional radiology, artificial intelligence, human–AI interaction, decision support, procedural verification, narrative review

## Abstract

Traditional evaluations of artificial intelligence (AI) systems in the dynamic, operator-dependent, and time-sensitive field of interventional radiology (IR), focusing solely on algorithmic performance, often fail to capture their real-world clinical impact. This narrative review aims to provide an overview of the current state of the art of AI integration in IR through human–AI interaction (HAI), while offering a critical perspective on their clinical integration, limitations, and future directions. A comprehensive survey of recent literature was performed, focusing on AI applications across procedural phases. The review emphasizes systems providing decision support, real-time procedural verification, and immersive interfaces (augmented and virtual reality), while critically evaluating determinants of effective clinical adoption. AI has shown preliminary potential to support operator performance in selected interventional radiology tasks, although most applications remain experimental, retrospective, or evaluated in phantom or preclinical settings. Potential benefits include structuring uncertainty in patient selection and procedural planning, supporting assessment of device positioning and treatment outcomes, and integrating AI-derived outputs into the operator’s spatial field through immersive technologies. The clinical utility of these systems appears to be influenced by human–AI interaction, with interpretability, workflow integration, and trust calibration representing key determinants of effective use beyond algorithmic accuracy alone. The potential value of AI in interventional radiology appears to derive from its integration into human decision-making rather than from standalone predictive performance alone. A human-centered, interaction-based model supports understanding current applications, address challenges, and guide the development of adaptive, real-time systems for dynamic procedural environments.

## 1. Introduction

The integration of artificial intelligence (AI) into interventional radiology (IR) may contribute to changes in how procedural decisions are planned, executed, and evaluated [1,2,3,4,5]. IR is a medical specialty that uses image-guided minimally invasive procedures for the diagnosis and treatment of a wide range of oncologic, vascular, and non-vascular diseases. Procedures are typically performed under fluoroscopic, ultrasound, CT, or cone-beam CT guidance and require continuous integration of imaging information, device manipulation, and real-time clinical decision-making. These characteristics distinguish IR from diagnostic radiology and make it a particularly relevant environment for studying human–AI interaction (HAI) [1,2,3,4,5]. IR is inherently procedural, time-sensitive, and operator-dependent [5]. In this context, the value of AI cannot be fully understood through an isolated evaluation of algorithmic performance [4,5]. Rather, it must be framed within the interaction between the clinician and the system [6].

Human–AI interaction may therefore provide a useful conceptual framework for evaluating AI applications in interventional radiology [6,7,8]. Core system properties, such as reliability, validation, and predictive performance, do not function as independent domains. In IR, they acquire clinical relevance only through their integration into the physician’s real-time decision-making process [9,10,11,12]. From this perspective, AI is not merely a computational tool but a component of a socio-technical system in which meaning, trust, and utility emerge through use [13].

Within this framework, the present narrative review aims to provide an overview of the current state of the art of advanced AI systems in interventional radiology, while offering a critical perspective on their clinical integration, limitations, and future directions [14,15,16]. As this is a narrative review, a formal systematic review methodology was not applied. Nevertheless, a structured literature search was performed using PubMed, Scopus, and Web of Science databases. Search terms included combinations of “artificial intelligence”, “machine learning”, “deep learning”, “human–AI interaction”, “interventional radiology”, “augmented reality”, “virtual reality”, “decision support”, and “procedural guidance”. Priority was given to recent studies, review articles, position papers, and original investigations relevant to clinical applications and HAI in IR.

The contribution of this review lies in its human–AI-interaction-centered perspective. This perspective emphasizes how factors such as interpretability, workflow integration, and trust influence the clinical adoption of AI systems.

## 2. Conceptual Framework

### 2.1. Human–AI Interaction as an Overarching Framework

Understanding the role of AI in IR may require a broader human-centered perspective [15]. The commonly adopted technology-centered perspective is replaced by a human-centered, interaction-based approach [17,18,19,20,21,22]. Traditional methods often assess AI systems as isolated entities, focusing on algorithmic performance metrics such as accuracy and predictive power [23,24,25]. However, in interventional radiology, where decision-making is dynamic, context-dependent, and time-critical, such an approach may be insufficient when considered alone [14,15,16]. Instead, AI systems should be conceptualized as components of a socio-technical system, in which their clinical value emerges through interaction with the operator [26,27,28]. In IR, AI performance is inherently linked to how human users perceive, interpret, and act based on the information [22,29,30]. Within this framework, human–AI interaction serves as the central axis for defining and assessing essential system properties [19,20,21]. For instance, reliability is not solely determined by statistical accuracy; it also depends on consistency, interpretability, and the calibration of trust during clinical use [22,29]. Similarly, predictive outputs acquire clinical meaning only when they are integrated into the physician’s reasoning process and translated into actionable decisions, as highlighted by Buijs et al., who show that the clinical impact of AI in radiology emerges primarily through its integration into clinical processes and workflow rather than through standalone algorithmic performance [31]. According to the literature, clinicians rarely passively accept AI recommendations/predictions. Interventional radiologists are more likely to engage in selective integration and contextual interpretation [32,33,34]. In IR, AI systems must advise interpretability and responsivity [2,4,14]. Procedural variability and real-time constraints reinforce the need for AI systems that are not only accurate but also usable, interpretable, and responsive [4,14,15,16]. This is the conceptual foundation for the present review [35]. Accordingly, the following sections will examine reliability, validation, and predictive modeling as interdependent dimensions of human–AI interaction within interventional radiology practice [36,37,38].

### 2.2. Interaction Dynamics in Clinical Practice

Human–AI interaction in interventional radiology is a dynamic and context-dependent form of decision-making [4,14,35]. Interventional radiologists actively engage with AI systems, integrating their outputs into procedural reasoning. This interaction can be broadly conceptualized across three recurring patterns: acceptance, rejection, and negotiated integration [35,37].

Acceptance occurs when AI outputs are directly incorporated into decision-making. This happens when system recommendations are consistent with clinical expectations, are presented clearly, and align with procedural goals. In such cases, AI can streamline decision-making, reduce uncertainty, and improve efficiency [39]. For example, AI-assisted image guidance or automated lesion detection tools are more readily adopted when they confirm operator expectations and reduce cognitive load during procedures [40,41].

Rejection, conversely, arises when clinicians disregard AI outputs. This may occur due to perceived inconsistency with clinical judgment, lack of transparency, or poor integration within the workflow [42]. For instance, studies on AI deployment in clinical settings show that lack of transparency and workflow misalignment are key drivers of non-use or active rejection by clinicians [43,44,45,46].

**Figure 1:**
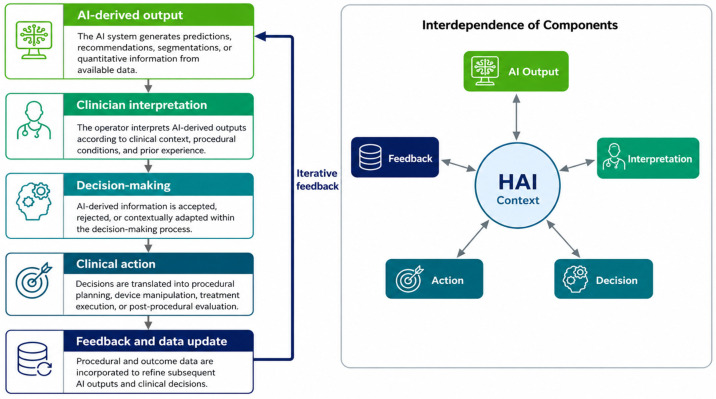
Conceptual framework of human–AI interaction (HAI) in interventional radiology. The left panel illustrates the typical workflow through which AI-derived outputs are interpreted by the clinician, integrated into the decision-making process, translated into clinical actions, and subsequently refined through feedback and data updates. The right panel highlights the interdependent and dynamic nature of AI outputs, clinician interpretation, decision-making, clinical actions, and feedback.

However, the most prevalent interaction pattern is negotiated integration. In this mode, clinicians neither fully accept nor reject AI outputs; instead, they interpret them, considering contextual factors such as anatomical variability, procedural feedback, and individual experience. AI outputs become one element within a broader cognitive process, contributing to but not determining the final decision [47]. This type of interaction aligns with evidence that clinicians dynamically adapt AI recommendations, combining them with experiential knowledge and situational awareness [48,49,50,51,52]. This negotiation process is particularly relevant in interventional radiology, where procedural conditions evolve in real time, and decision-making must remain flexible [2,4,14,15]. It highlights that the clinical value of AI does not reside solely in its outputs, but in how those outputs are incorporated into human reasoning. Human–AI interaction in interventional radiology can be conceptualized as a dynamic and iterative process, as illustrated in Figure 1.

### 2.3. Determinants of Effective Human–AI Interaction

The effectiveness of human–AI interaction in interventional radiology is shaped by multiple interrelated factors that influence how AI outputs are perceived, interpreted, and utilized during image-guided procedures. These determinants operate at the interface between system design and human cognition.

Interface design plays a central role in mediating interaction in interventional radiology. AI outputs must therefore be presented in a manner that is immediately interpretable and spatially aligned with procedural anatomy. Techniques such as image overlays, real-time segmentation, trajectory visualization, and needle path guidance can enhance usability by embedding AI outputs directly within the interventional field [53]. Equally important is workflow integration. AI systems that require additional steps, disrupt procedural flow, or operate outside existing imaging and navigation platforms are less likely to be adopted [9]. In IR, where procedures depend on fluid coordination among imaging, device manipulation, and decision-making, effective systems integrate seamlessly into angiographic suites and navigation environments, supporting rather than interrupting operator actions [54].

Interventional radiology is inherently time-sensitive, with decisions often made under strict temporal constraints. The utility of AI systems therefore depends heavily on their ability to deliver outputs with minimal latency [55]. In intra-procedural settings, even slight delays can compromise usability, particularly in tasks such as needle placement, catheter navigation, or embolization guidance [56]. Real-time or near-real-time performance is a fundamental condition for effective interaction. Moreover, temporal alignment between AI outputs and procedural steps is critical: information must be delivered at the precise moment it is needed to support operator decisions [56].

The interventional environment imposes substantial cognitive demands on the operator. AI systems must therefore be designed to minimize additional cognitive burden [57,58]. Excessive information, poorly organized outputs, or non-intuitive interfaces can increase cognitive load and interfere with procedural performance [59,60]. Conversely, systems that present concise, context-aware, and spatially relevant information, can enhance situational awareness without distracting the operator. In this setting, usability is directly linked to procedural safety and efficiency [61,62,63,64].

Explainability is a key factor influencing clinician trust in AI systems [65,66]. Operators must be able to rapidly understand and assess the basis of AI outputs, especially when these inform critical intra-procedural decisions [65]. However, explainability must be balanced with usability. Highly detailed explanations may be impractical during procedures, while insufficient transparency can lead to mistrust or rejection [67,68]. Effective systems provide actionable transparency, offering interpretable cues without interrupting workflow [66,67,68]. Trust in AI systems is dynamic and evolves through repeated interaction. Clinicians continuously calibrate their reliance levels based on system consistency, predictability, and behavior under varying procedural conditions [69].

## 3. AI for Decision Support

AI is increasingly used in IR to support clinical decision-making across all procedural phases. Its role is not to replace the operator but to provide structured, data-driven information that reduces uncertainty and improves consistency [70,71]. In such settings, human decision-making is inherently probabilistic and experience dependent. AI systems can augment this process by identifying patterns that are not easily detectable through conventional analysis [72,73]. These systems decline at various phases in IR.

In the pre-procedural phase, AI is primarily applied to patient selection, risk stratification, and procedural planning [74,75]. These applications are particularly developed in oncologic interventional radiology, where treatment decisions are influenced by both tumor characteristics and patient-specific factors [37]. For example, radiomics and machine learning models have been extensively studied in hepatocellular carcinoma (HCC) [76,77]. Several works have demonstrated that models combining CT-derived texture features with clinical variables can predict response to transarterial chemoembolization (TACE) [78,79,80,81,82]. AI-based models for TACE response and outcome prediction generally demonstrated moderate-to-high predictive performance, with reported AUC values commonly ranging from approximately 0.75 to 0.95 depending on the endpoint, model architecture, and validation strategy [78,79,80,81,82]. However, direct quantitative comparison remains challenging because of substantial heterogeneity in patient populations, imaging protocols, outcome definitions, and validation methods. In some studies, these models have outperformed traditional staging systems, such as the BCLC or Child–Pugh classification [83,84], particularly in identifying patients with an intermediate prognosis who may benefit from treatment. Beyond HCC, similar approaches have been applied to other domains. In percutaneous ablation, predictive models have been developed to estimate the risk of incomplete treatment based on lesion size, location, and proximity to vessels [85,86,87,88]. In vascular interventions, machine learning models have been used to predict complications such as post-contrast acute kidney injury, including contrast-associated acute kidney injury or post-contrast acute kidney injury (CA-AKI/PC-AKI), as well as access-site bleeding [89,90]. These models typically integrate clinical, laboratory, and imaging data, offering a more comprehensive assessment than single-parameter risk scores [89,90,91,92,93]. Despite these advances, the outputs of these systems remain probabilistic. They do not provide definitive answers but rather estimates that must be interpreted [94]. In practice, clinicians combine these predictions with procedural considerations, such as the accessibility of the target lesion, the expected procedure duration, and the operator’s experience [95]. This results in a decision-making process in which AI helps structure uncertainty rather than eliminate it.

In the intra-procedural phase, AI primarily assists with image interpretation, target identification, and trajectory planning [96,97]. This is particularly relevant in CT-guided and cone-beam CT (CBCT)-guided interventions, where accurate spatial understanding is essential [4,15,96]. Several commercial platforms already incorporate elements of decision support. Systems such as Syngo Needle Guidance (Siemens Healthineers) and XperGuide (Philips) enable operators to plan needle trajectories using 3D imaging [96,97,98]. These systems provide visual overlays that indicate the optimal path to the target while avoiding critical structures. More recent developments include AI-based segmentation tools that automatically identify lesions, vessels, and organs at risk, reducing the need for manual contouring [99]. In angiographic procedures, deep learning models have been developed for automatic vessel detection and enhancement [100,101,102,103]. These systems can improve visualization of complex vascular anatomy, particularly in low-contrast conditions [104,105,106]. Some studies have reported reductions in fluoroscopy time and contrast use when AI-assisted navigation is used, suggesting potential benefits for procedural efficiency and safety [15,107,108,109,110].

In addition to pre-procedural predictions, there is growing interest in AI systems capable of providing post-procedural estimates given intra-procedural data [111,112,113,114,115,116]. These models aim to support dynamic decision-making by integrating real-time data from imaging, devices, and procedural parameters [114]. One of the most studied applications is thermal ablation [117]. Several models have been developed to predict the extent of the ablation zone based on energy delivery, tissue characteristics, and probe position [117,118,119,120,121]. These systems can generate volumetric estimates of expected necrosis, allowing operators to adjust probe placement or energy settings during the procedure [117,118,119,120]. Early studies have shown moderate to good agreement between predicted and observed ablation volumes, although variability remains, particularly in heterogeneous tissues. In embolization procedures, experimental approaches have explored the use of AI to predict treatment endpoints [121]. By analyzing intra-procedural imaging patterns, such as contrast distribution or flow dynamics, these systems aim to estimate the completeness of embolization [122,123,124,125,126]. While still in early stages, such models suggest a shift toward adaptive systems that respond to evolving procedural conditions [127,128]. A key limitation of these approaches is the potential increase in cognitive load. Continuous predictive outputs can be useful, but only if they are concise and directly actionable [129,130].

Overall, AI-based decision support systems in interventional radiology have the potential to improve several aspects of clinical practice [124,125]. These include more accurate patient selection, better procedural planning, enhanced targeting precision, and more personalized treatment strategies. However, clinical impact is not determined solely by predictive performance. Multiple studies have shown that adoption depends on factors such as interpretability, timing, and integration into workflow [4,131,132].

## 4. AI for Procedural Verification

AI is increasingly used in interventional radiology to verify procedures [15,55]. In this context, its role differs from decision support. Rather than informing what should be done, AI is used to assess what has been done [55]. This includes confirming device positioning, evaluating treatment completeness, and detecting deviations from expected procedural outcomes [133]. Verification functions are particularly relevant in IR due to the limited direct visualization of targets and the reliance on indirect imaging feedback [134]. Errors in positioning or incomplete treatment may not be immediately evident. AI systems can provide an additional layer of control by analyzing imaging data in real time and highlighting discrepancies that may otherwise go unnoticed [132].

One of the most immediate applications of AI in verification is the assessment of device positioning. Accurate placement of needles, catheters, or guidewires is critical in most interventional procedures. Small deviations can significantly affect treatment efficacy and complication rates [134].

AI-based image analysis tools have been developed to automatically detect and track devices within CT, CBCT, or fluoroscopic images. These systems can identify the spatial relationship between the device tip and the target lesion, providing real-time feedback to the operator. In CT-guided interventions, experimental studies have explored deep learning-based needle detection, tracking, and shape prediction approaches to support device localization during procedures [135]. However, current evidence does not yet demonstrate clinically validated real-time verification systems capable of consistently achieving sub-millimetric needle-tip localization accuracy in routine interventional radiology practice. In endovascular procedures, AI-assisted vessel tracking and catheter detection have also been explored. These systems can enhance visualization of the catheter path and identify unintended deviations, particularly in complex vascular territories [136,137]. Early studies suggest that such tools may reduce navigation errors and improve procedural precision, though widespread clinical adoption remains limited.

Beyond device positioning, AI can be used to assess whether a therapeutic objective has been achieved. This is particularly relevant in procedures such as thermal ablation and embolization, where treatment success is not always immediately visible [138,139]. In thermal ablation, post-procedural imaging is typically used to evaluate the extent of necrosis [120]. AI-based segmentation tools can automatically delineate the ablation zone and compare it with the original tumor volume. Some systems can quantify margins and identify residual viable tissue [120,121]. In embolization procedures, verification is more complex. Treatment success is often inferred from changes in blood flow or contrast distribution [140]. AI models have been developed to analyze angiographic sequences and detect patterns associated with complete or incomplete embolization [141]. For example, AI- and radiomics-based approaches have been investigated for prognostic prediction and treatment-response assessment after TACE in hepatocellular carcinoma, with promising but still preliminary results [141].

A more advanced application of AI in verification is real-time feedback during procedures. In this setting, AI systems continuously analyze incoming data and identify deviations from expected patterns [75,142].

Examples include detecting needle deflection during insertion, identifying non-target embolization, or recognizing suboptimal probe placement during ablation [142,143]. Some experimental systems use continuous image analysis to alert the operator when the device trajectory deviates from the planned path [144]. Others analyze contrast flow patterns to detect unintended distribution of embolic material [144,145,146,147,148,149]. In ultrasound-guided interventions, AI has been used to improve needle visualization and detect misalignment between the needle and the imaging plane [150]. These systems can provide immediate feedback, which is particularly useful in procedures where visualization is operator-dependent [151].

The main challenge of real-time verification systems is balancing sensitivity and specificity. Excessive alerts may lead to alarm fatigue, while insufficient sensitivity may fail to detect critical errors. Therefore, these systems must be carefully calibrated to provide clinically relevant feedback without disrupting workflow [145,146,147,148,149,150,151].

## 5. Immersive Interfaces in Interventional Radiology: A Transversal Layer of Human–AI Interaction

Immersive technologies, including augmented reality (AR), virtual reality (VR), and extended reality (XR), represent a transversal layer of human–AI interaction in interventional radiology [152,153]. Unlike decision support and verification systems, which are typically associated with specific procedural phases, these technologies operate across the entire clinical continuum [154]. Their potential contribution lies not only in generating new predictions, but also in modifying how AI-derived information may be perceived, interpreted, and integrated into clinical action [155,156]. By embedding computational outputs directly into the operator’s visual and spatial field, immersive interfaces reduce the separation between data and decision [155,156]. This transformation shifts interaction from a screen-based paradigm to a spatially integrated form of cognition, in which AI outputs become part of the procedural environment rather than external inputs requiring interpretation [55].

In the pre-procedural phase, VR and AR systems are primarily used for planning and simulation [152,153,154]. AI-based models derived from CT, MRI, or cone-beam CT can be reconstructed into three-dimensional representations of patient-specific anatomy [97]. These models can then be explored within immersive environments, allowing operators to visualize complex spatial relationships with greater precision than conventional 2D or static 3D reconstructions [157,158,159,160,161,162]. This approach is particularly relevant in oncologic and vascular interventions [163]. For example, in liver-directed therapies, VR-based visualization of tumor location relative to vascular structures has been shown to improve planning accuracy and operator confidence [164]. Similarly, in complex aortic or peripheral vascular procedures, immersive models enable detailed assessment of vessel geometry, branching patterns, and access routes [165,166,167,168,169]. From a human–AI interaction perspective, immersive planning reduces cognitive load associated with mental reconstruction of anatomy. Instead of translating multiple image slices into a coherent spatial model, operators interact directly with AI-generated representations [170]. This facilitates intuitive understanding and may support more consistent procedural strategies, particularly in anatomically complex or borderline cases [170].

During procedures, AR-based systems enable real-time integration of AI outputs into the operative field. Information such as segmented lesions, vascular structures, risk zones, or planned trajectories can be overlaid onto live imaging modalities, including fluoroscopy, ultrasound, or CT [154,155]. In some systems, head-mounted displays or projection-based interfaces allow direct visualization of these overlays within the operator’s field of view [154,155]. This spatial alignment addresses a key limitation of conventional interfaces: the need to continuously switch between imaging screens and the procedural field. By co-registering virtual information with real anatomy, AR systems reduce the cognitive effort required to map image-based data onto physical actions [157,164]. This is particularly relevant in tasks such as needle placement, catheter navigation, and ablation targeting, where precision depends on accurate spatial interpretation [164]. Clinical studies have reported improvements in targeting accuracy and reductions in procedure time with AR-assisted navigation in percutaneous interventions, although results remain variable and dependent on system accuracy and workflow integration [171,172,173]. In ultrasound-guided procedures, AR overlays have been used to enhance needle visualization and improve alignment with the imaging plane, addressing known limitations of operator-dependent imaging [156]. Within the HAI framework, these systems enable a more direct form of interaction. AI outputs are no longer passively observed but actively engaged within the procedural context. This supports a form of interaction that is continuous, context-aware, and tightly coupled with operator actions.

In the post-procedural phase, immersive technologies support evaluation, verification, and training. AI-based segmentation of treatment zones, such as ablation volumes or embolized territories, can be visualized in three-dimensional environments and directly compared with pre-procedural targets [174]. This enables more precise assessment of treatment margins and spatial completeness than conventional slice-based review. In addition, VR environments provide a platform for retrospective procedural analysis. Operators can review procedural steps, device trajectories, and outcomes in an interactive setting. This is particularly relevant for training and quality improvement, where understanding the spatial dynamics of a procedure is critical [175]. Simulation-based training platforms integrating AI and VR have also been developed for interventional radiology. These systems allow rehearsal of procedures using patient-specific data, enabling operators to anticipate technical challenges and refine strategies before actual intervention [176,177]. Early evidence suggests that such approaches may improve procedural performance and reduce variability, although standardized evaluation metrics remain limited. From an interaction standpoint, post-procedural immersive environments extend the role of AI beyond real-time decision-making. They support reflective analysis and learning, contributing to the continuous calibration of operator understanding and trust in AI systems [174,175,176].

Immersive interfaces may modify the nature of human–AI interaction in interventional radiology [55]. This has direct implications for usability, efficiency, and trust calibration [15,55]. In contrast to traditional interfaces, where AI outputs are presented as abstract data requiring interpretation, immersive systems provide contextually aligned information that can be directly acted upon. This alignment supports faster decision-making and may reduce the risk of misinterpretation, particularly in time-sensitive scenarios [178]. At the same time, these systems introduce new challenges. Accurate spatial registration between virtual and real anatomy remains technically demanding, particularly in the presence of organ motion or deformation. Latency and hardware constraints may affect real-time usability. Furthermore, integration into existing workflows and imaging platforms is essential to avoid disruption of procedural flow [179]. Ultimately, the clinical impact of AR and VR technologies depends not only on their technical performance but on their ability to align with the cognitive and operational demands of interventional radiology [171,172,173]. Within the HAI framework, their value lies in transforming how AI is experienced by the operator-shifting from indirect interpretation to direct, spatially embedded interaction. The information presented above is schematically summarized in Table 1.

## 6. Limitations and Challenges

Despite the growing body of evidence supporting the integration of artificial intelligence in interventional radiology, several limitations remain that constrain its current clinical impact. Importantly, within a human–AI interaction framework, these limitations cannot be interpreted as purely technical shortcomings but must be understood as systemic constraints emerging at the interface between algorithms, users, and clinical environments [4,15,55]. A primary limitation of current AI systems in IR is their limited robustness across heterogeneous clinical settings. A practical limitation is the lack of large multicenter interventional radiology datasets with standardized procedural annotations and clinically meaningful outcome measures. Compared with diagnostic radiology, IR datasets are more heterogeneous and include dynamic procedural variables, device manipulation, and operator-dependent factors, which currently limit reproducibility and external validation across institutions [15,176]. Most models are developed and validated on retrospective datasets obtained from single centers, often under controlled conditions. As a result, their performance may degrade when applied to different imaging protocols, devices, or patient populations [14,76,174,175]. This issue is particularly relevant in interventional radiology, where intra-procedural variability is high and imaging conditions are less standardized than in diagnostic workflows [176]. As highlighted in recent radiology AI literature, model performance is highly sensitive to domain shift and data heterogeneity, which can significantly affect reliability in real-world applications [174,175]. Furthermore, intra-procedural environments introduce additional sources of variability, including motion, artifacts, and device-related distortions [180]. These factors remain challenging for current AI models, particularly in real-time applications. A critical gap in the current literature concerns the validation of AI systems at the level of clinical interaction. While many studies report high predictive performance using metrics such as AUC or accuracy, relatively few assess the actual impact of these systems on decision-making, procedural outcomes, or workflow efficiency [178]. Moreover, prospective studies evaluating the effect of AI systems on clinically relevant endpoints, such as technical success, complication rates, radiation exposure, procedure duration, and workflow integration, remain limited [31,55,181,182,183]. As emphasized by Topol and others, the translation of AI from experimental settings to clinical practice requires rigorous prospective validation and evaluation of real-world utility [15,17,179]. In interventional radiology, this limitation is even more pronounced. The effectiveness of AI systems depends not only on predictive accuracy but also on how outputs are interpreted and acted upon during procedures [181]. However, most validation frameworks do not capture this interactional dimension as noted by Buijs et al. [31]. This suggests that current validation paradigms may be insufficient, as they fail to account for human–AI collaboration as a determinant of clinical outcomes [182,183,184]. The integration of AI into interventional practice introduces additional cognitive and behavioral challenges. Interventional radiologists operate in high-pressure environments characterized by time constraints, multitasking, and continuous decision-making. In this context, poorly designed AI systems may increase cognitive load rather than reduce it. Excessive or poorly structured outputs can interfere with situational awareness, while unclear or non-intuitive interfaces may lead to underutilization or misuse [185]. Moreover, behavioral phenomena such as automation bias and algorithm aversion can further complicate interaction. As described in human–AI interaction literature, clinicians may either over-rely on AI recommendations or systematically disregard them depending on trust calibration and prior experience [186]. This variability highlights that human factors are not secondary considerations but central determinants of system effectiveness. Beyond technical and human factors, several structural barriers limit the implementation of AI in interventional radiology. These include challenges related to interoperability with existing imaging platforms, integration into clinical workflows, and compliance with regulatory frameworks. In addition, medico-legal considerations remain unresolved [187]. Ethical concerns also arise regarding transparency, bias, and equitable access to AI technologies. As emphasized in recent policy discussions, the deployment of AI in healthcare requires not only technical validation but also governance frameworks that ensure safety, accountability, and fairness [188,189,190]. To further contextualize the current level of evidence supporting AI applications in interventional radiology, representative studies are summarized in Table 2.

## 7. Future Directions

The future development of AI in interventional radiology is likely to be characterized by a shift from isolated predictive tools toward adaptive, interaction-centered systems [2,4]. In this evolution, the focus moves from algorithmic performance to the design of systems that effectively support human decision-making in dynamic procedural environments [14,15]. Current AI systems are largely static, providing predefined outputs based on fixed models. Future priorities should include multicenter data collection, standardized procedural annotation, prospective clinical validation, and integration of AI systems into existing angiographic, CT, ultrasound, and navigation platforms. In addition, future studies should incorporate usability, cognitive load, trust calibration, and workflow-related endpoints as part of HAI evaluation. Future systems may increasingly incorporate adaptive capabilities that respond to procedural context and evolving intra-procedural data [2,14,15].

As suggested in emerging HAI research, adaptive systems may improve both usability and trust by aligning AI behavior with individual operator needs [194]. A key direction is the development of closed-loop systems capable of integrating real-time data, generating predictions, and updating recommendations dynamically during procedures [195,196,197,198]. Such systems would move beyond static decision support, enabling continuous interaction between data acquisition, prediction, and action [198,199]. In interventional radiology, this could translate into real-time adjustment of needle trajectories, ablation parameters, or embolization strategies based on ongoing feedback [37,56,128]. Finally, future AI systems will likely integrate multiple data sources, including imaging, clinical records, procedural parameters, and device-generated data. This multimodal approach may enable more comprehensive and context-aware decision support [200,201,202]. Emerging generative AI ecosystems, including multimodal foundation models, large language models, and agentic AI systems, may further influence human–AI collaboration in IR. Potential applications include procedural planning support, workflow optimization, automated documentation, multimodal decision support, and integration of imaging, clinical, and procedural information within unified interaction frameworks. However, their role in routine interventional radiology practice remains largely unexplored and requires prospective evaluation [11,30,198]. The integration of AI with immersive interfaces such as augmented reality and virtual reality represents a particularly promising direction [162,163,164]. By embedding predictive outputs within the operator’s spatial field, these systems may enable more intuitive and efficient interaction [201]. Future developments may combine real-time prediction, spatial visualization, and procedural guidance into unified platforms. As noted in recent studies, such convergence has the potential to transform decision-making from a screen-based process to a spatially integrated experience [162,203,204,205].

## 8. Conclusions

The integration of AI into interventional radiology represents a promising but still evolving field moving from technology-centered tools toward more interaction-centered systems [203]. Within this framework, the clinical value of AI does not reside solely in its predictive performance but emerges through its integration into human decision-making processes [201,205,206,207,208]. This review has highlighted how reliability, validation, and prediction should be understood as interdependent dimensions of human–AI interaction, shaping how AI systems are perceived, interpreted, and utilized in procedural contexts. Current applications suggest promising potential across decision support, procedural verification, and immersive interaction; however, many remain experimental, retrospective, or insufficiently validated in prospective clinical settings. However, their clinical impact remains contingent on factors such as usability, workflow integration, and trust calibration. Future developments may increasingly focus on adaptive, real-time, and human-centered systems designed to support dynamic interaction in complex procedural environments. Ultimately, the integration of AI in interventional radiology will likely depend not only on advances in algorithmic performance, but also on prospective clinical validation and on the ability to design systems that align with the cognitive, procedural, and organizational realities of clinical practice.

## Figures and Tables

**Table 1:** AI applications in interventional radiology across procedural phases and functional roles.

Procedural Phase	AI Application	Functional Role	Interaction Modality	Clinical Added Value
Pre-procedural	Risk stratification, patient selection, procedural planning	Decision support	Probabilistic prediction integrated into clinical reasoning	Reduction in uncertainty, improved patient selection and planning consistency
Intra-procedural	Image guidance, target segmentation, needle trajectory planning	Real-time decision support	Continuous human–AI interaction with dynamic feedback	Increased targeting accuracy, reduced cognitive load, improved procedural efficiency
	Device tracking, treatment monitoring, endpoint prediction	Procedural verification	Real-time feedback and deviation detection	Early error detection, improved treatment completeness
Post-procedural	Ablation zone assessment, outcome prediction	Outcome verification and evaluation	Retrospective interpretation of AI outputs	Objective assessment of treatment success and margins
Transversal (all phases)	Augmented reality (AR), virtual reality (VR), extended reality (XR)	Immersive interaction layer	Spatial integration of AI outputs into operator field	Enhanced spatial understanding, reduced cognitive load, improved workflow integration

**Table 2:** Representative AI applications in interventional radiology: study characteristics, validation strategies, clinical maturity, human–AI interaction considerations, and current limitations.

Study	AI Task	IR Procedure/ Application	Study Type	Sample Size	Validation Type	Primary Metric	Clinical Endpoint	HAI/Usability Assessment	Clinical Maturity Level	Main Limitations
**Abajian et al., 2018 [138]**	Supervised ML prediction of treatment response	Intra-arterial therapy/TACE for HCC	Retrospective proof-of-concept/methodological study	36 patients	Leave-one-out internal cross-validation	AUC/accuracy	Imaging-based tumor response according to qEASL	-	Proof of concept/technical validation	Small cohort; no external validation; limited assessment of clinical utility or workflow integration
**Mamone et al., 2024 [191]**	CT radiomics/ML outcome prediction	TIPS creation	Retrospective single-center cohort	76 patients	Internal model evaluation	AUROC: 0.767 for clinical response; 0.757 for 6-month survival; 0.744 for grade ≥ 2 HE	Hepatic encephalopathy, clinical response, and 6-month survival after TIPS	-	Technical validation	Single-center retrospective study; small cohort; no external validation; no prospective decision-impact analysis
**Ueda et al., 2025 [190]**	Automated tumor-feeder detection	Selective TACE for HCC using angio-CT	Retrospective clinical workflow study	74 patients; 107 HCC tumors; 114 feeding arteries	Comparison with radiologist interpretation and procedural reference standard	Sensitivity 90.4%; PPV 90.4%	Feeding artery identification; technical success defined by complete lipiodol uptake	Partial: software integrated into workflow; analysis generally completed in <5 min	Clinical feasibility/early clinical validation	Retrospective; single-center; false-positive and missed feeders; failures more frequent in complex/repeated TACE cases
**Abdelsalam et al., 2022 [192]**	Automated feeder detection/procedural guidance	CBCT-guided TACE with EmboGuide for HCC	Prospective comparative non-randomized study	Study group: 44 patients/57 lesions; control group: 41 patients/55 lesions	Clinical comparison with control group lacking AFD software	Feeder agreement rate: 91.2%	Residual non-embolized tumor area at 1 month; radiation exposure; procedural efficiency	Partial: applied during real TACE workflow by interventional radiologist	Clinical feasibility/comparative clinical evaluation	Non-randomized design; limited sample size; single procedural context; operator and institutional effects possible
**Bartnik et al., 2024 [193]**	Automated DL segmentation + radiomics/survival prediction	Pre-TACE assessment in unresectable HCC	Retrospective single-center cohort	252 patients; 734 TACE procedures	Repeated cross-validation: 10 × eightfold for OS; 10 × fivefold for PFS	C-index: 0.640 for OS; 0.713 for PFS	Overall survival and progression-free survival after TACE	Indirect: no radiologist input required for multi-organ segmentation	Technical validation/translational research	Single-center retrospective study; no prospective validation; clinical decision impact not tested
**Grube et al., 2024 [150]**	3D needle-tip localization	Ultrasound-guided needle navigation	Experimental preclinical study	Large dataset of low-resolution US volumes acquired in water and chicken liver tissue	Internal experimental validation; comparison with conventional needle segmentation	Mean position error	Needle-tip localization accuracy for real-time navigation	-	Preclinical technical validation	Phantom/ex vivo setting; no patients; no procedural clinical endpoints; no usability or operator-impact assessment

## Data Availability

The original contributions presented in this study are included in the article. Further inquiries can be directed to the corresponding author.

## Abbreviations

The following abbreviations are used in this manuscript:

BEVAR	Branched Endovascular Aneurysm Repair
CNR	Contrast-to-Noise Ratio
CTA	Computed Tomography Angiography
DECT	Dual-Energy Computed Tomography
DECTA	Dual-Energy Computed Tomography Angiography
DSA	Digital Subtraction Angiography
